# Breast lipoma with central fat necrosis: case report

**DOI:** 10.11604/pamj.2016.25.235.10935

**Published:** 2016-12-13

**Authors:** Mohamed Reda Bouroumane, Reda Khalil, Hind Khalil, Hicham Jalal

**Affiliations:** 1 Department of Radiology, University Hospital Mohammed VI of Marrakesh, Morocco

**Keywords:** Fat necrosis, lipoma, mammography, eggshell, calcifications, breast

## Abstract

Fat necrosis of the breast is a benign non-suppurative inflammatory process of adipose tissue that most commonly occurs as the result of minor breast trauma. We present a case of a 40-years-old female with fat necrosis in a breast lipoma. She presented with an overlapping mass on the lateral quadrants. Mammography showed Well delineated radiolucent mass with peripheral “egg-shell” calcifications, that appeared an hypoechoic mass with posterior shadowing on ultrasonography. A history of accidental trauma raises the suspicion of fat necrosis. Pathology is necessary when radiological findings simulate malignancy.

## Introduction

Fat necrosis of the breast is a benign non-suppurative inflammatory process of adipose tissue that most commonly occurs as the result of minor breast trauma. The occurrence of fat necrosis in lipomas is reported to be rare and so are the imaging findings. Here, we present a case of fat necrosis in a breast lipoma in order to focus on the fundamental role of imaging techniques in the diagnosis.

## Patient and observation

**Figure 1:**
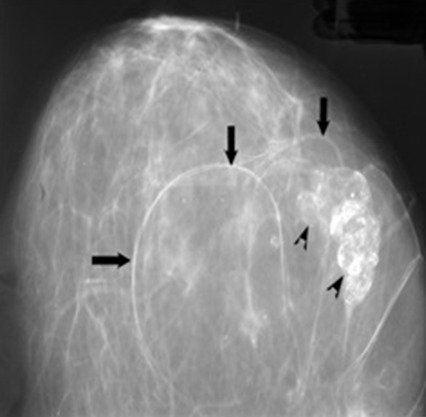
Lipoma in a 40-year-old woman with a palpable lump in the right breast; Cranio caudal mammogram of the right breast: giant radiolucent mass, with well delineated thin lobulated capsule (arrows) overlapping the lateral quadrants on 9 O’clock; this formation is the seat of an ill defined irregular mass-lik area with coarse and eggshell (arrowheads) calcifications within

**Figure 2:**
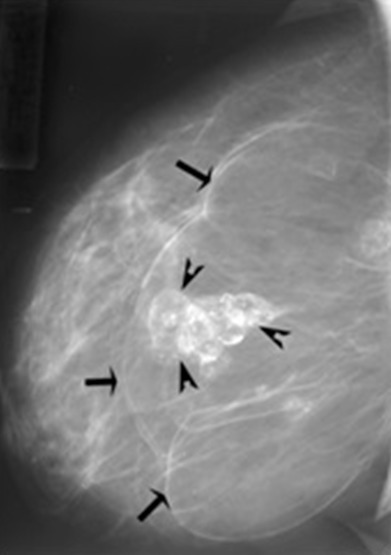
Lipoma in a 40-year-old woman with a palpable lump in the right breast; Mediolateral mammogram of the right breast:giant radiolucent mass, with well delineated thin lobulated capsule (arrows) overlapping the lateral quadrants on 9 O’clock (a;b). This formation is the seat of an ill defined irregular mass-like area with coarse and eggshell (arrowheads) calcifications within

**Figure 3:**
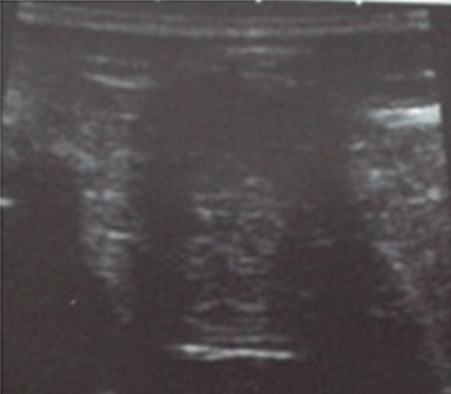
Lipoma in a 40-year-old woman with a palpable lump in the right breast; Ultrasound examination: ultrasonography showed a predominantly hypoechoic lesion with posterior shadowing

A 40-years-old female presented with 3-months history of right breast pain. Her past and family histories were unremarkable. Physical examination revealed an overlapping mass on the lateral quadrants of the right breast at 9 O'clock, firm and well defined, mobile, with no modification of the skin above and no axillary lymph nodes. Mammography showed a voluminous, well-delineated, multilobular and radiolucent mass, with thin peripheral capsule, overlapping the lateral quadrants of the right breast consistent with lipoma (Figure 1, Figure 2). Within, we noticed a central mass-like area showing irregular ill-defined margins with coarse, stippled and curvilinear calcifications creating the aspect of lucent “bubbles” or “eggshell calcifications” in the breast parenchyma, which corresponds to central fat necrosis (Figure 1, Figure 2). Ultrasonography (USG) showed a hypoechoic mass with well-defined lobulated margins and posterior shadowing consistent with central calcifications (Figure 3). Lipoma with central fat necrosis is considered; surgical excision of the mass and pathology examination confirmed the diagnosis.

## Discussion

The occurrence of fat necrosis in breast lipoma is uncommon, and few cases have been reported. A history of accidental trauma raises the suspicion of fat necrosis in a breast lump; the other common predisposing causes include surgery, radiation and anticoagulant therapy [1, 2]. The clinical presentation can vary from being clinically occult or usually painful, mobile breast lump without skin changes to a hard lump with skin changes highly suspicious for malignancy [1, 2]. Mammographic features of lipoma with central fat necrosis are characteristic. Lipoma appears as a round or oval radiolucent mass with well defined thin capsule. Mammography is the most accurate diagnostic tool in early fat necrosis and different features can be noted [1–4]: more often unique or multiple, round or oval, smooth-bordered lucent mass with a thin rim that may show eggshell calcifications. Fat-fluid level corresponds to oil and sero-sanguinous fluid layering. The benign lucent-centered calcification is a characteristic late stage feature. - Focally clustered, pleomorphic microcalcifications that are mammographically indistinguishable from those of malignancy; non-lucent focal mass with irregular contours with or without micro or macro-calcifications of variable size. It may show spiculated margins and cause retraction of the skin above simulating malignancy; numerous vascular calcifications with no opacity overhead. USG plays an important role in ruling out malignancy and suggesting the diagnosis of fat necrosis. It demonstrates a well circumscribed round or ovoid hypoechoic mass consistent with lipoma and a central heterogeneous mass-like area, seat of macro-calcifications with posterior acoustic shadowing [5–7]. The radiological appearance of lipoma with central fat necrosis of the breast associating radiological signs of both lipoma and fat necrosis is characteristic and may not require any additional work-up [8]. When any suspicious features for malignancy are noticed (dense or irregular mass, architectural distortion, or suspicious calcifications are found on mammogram) biopsy and pathology examination should be considered [9].

## Conclusion

The association of fat necrosis and lipoma is exceptional. Radiological features includes signs of both lipoma and fat necrosis. Pathology is necessary when radiological findings simulate malignancy.
